# Ultrasound dynamics during treatment of pulmonary and extra-pulmonary TB

**DOI:** 10.5588/ijtldopen.25.0608

**Published:** 2026-03-13

**Authors:** S.F. Weber, F. Tobian, M. Gaeddert, R. Wolf, K. Manten, D. Shankar, B. Thangakunam, B. Isaac, A.K. Dutta, N. Wetzstein, M.J.G.T. Vehreschild, I. Suárez, J. Rybniker, P. Wolf, F. Herth, S. Bélard, D.J. Christopher, C.M. Denkinger, the ALL POCUS TB Study Group

**Affiliations:** 1 Department of Infectious Disease and Tropical Medicine, Heidelberg University Hospital, Heidelberg, Germany;; 2 Department of Parasitology, Heidelberg University Hospital, Heidelberg, Germany;; 3 German Center for Infectious Disease Research (DZIF) partner site Heidelberg, Heidelberg, Germany;; 4 Department of Anesthesiology, Heidelberg University Hospital, Heidelberg, Germany;; 5 Department of Pulmonary Medicine, Christian Medical College Vellore, Vellore, India;; 6 Department of Clinical Gastroenterology, Christian Medical College Vellore, Vellore, India;; 7 Department II of Internal Medicine, Infectious Diseases, Goethe University Frankfurt, University Hospital Frankfurt, Frankfurt am Main, Germany;; 8 Department I of Internal Medicine, Division of Infectious Diseases, Medical Faculty and University Hospital of Cologne, University of Cologne, Cologne, Germany;; 9 German Center for Infectious Disease Research, DZIF Partner Site Cologne, Cologne, Germany;; 10 Department for Pneumology and Critical Care Medicine, Thoraxklinik Heidelberg, Heidelberg, Germany;; 11 Translational Lung Research Center Heidelberg, Heidelberg, Germany;; 12 Institute of Tropical Medicine, University of Tübingen, Tübingen, Germany;; 13 German Center for Infectious Disease Research, DZIF Partner Site Tübingen, Tübingen, Germany.

**Keywords:** tuberculosis, Germany, India, treatment monitoring, TB sequelae, paradoxical reaction, point-of-care ultrasound

## Abstract

**BACKGROUND:**

Point-of-care ultrasound (POCUS) offers an accessible tool for TB screening, particularly in resource-limited settings. Data on the longitudinal dynamics of sonographic pulmonary (PTB) and extra-pulmonary TB (EPTB) findings during anti-TB treatment (ATT) for potential therapy monitoring remain limited.

**METHODS:**

This secondary analysis of a prospective, multi-centre accuracy study of adults with presumed TB at one Indian and three German referral hospitals followed up participants undergoing ATT. Participants underwent lung (LUS) and extra-pulmonary ultrasound for pleural effusions, lymphadenopathy (peripheral, abdominal, internal mammary), ascites, and peritoneal pathology, before and during ATT. LUS findings were calculated as a score and compared between time-points.

**RESULTS:**

71 participants were enrolled from January 2022 to July 2023; most had pulmonary pathology (n = 68) or pleural effusion (n = 33). Median LUS scores declined consistently to 86% after 2–3 months of ATT compared to baseline. After 6–8 months, median LUS score was 47%, suggesting persistence of pathology. Pleural effusion showed improvements after 2–3 months in 30%, and peripheral lymphadenopathy persisted in 33% even after ATT completion.

**CONCLUSION:**

Sonographic findings in TB typically resolved with ATT but resolution may be incomplete even after 6 months. This study provides evidence supporting the potential role of ultrasound monitoring for the response of TB to ATT.

TB still causes an estimated 10.8 million new cases annually. Anti-TB treatment (ATT) is successful in 88% of new/relapse cases, but only in 78% of previously treated, 79% of HIV-positive, and 68% of multidrug-resistant (MDR) TB cases.^1^ Accurate and accessible tools are essential for treatment monitoring to identify individuals at risk of adverse treatment outcomes or inadequate treatment response, who require additional support or different therapeutic measures. Guidelines for TB treatment monitoring currently include repeated sputum cultures and clinical evaluation throughout ATT.^2–4^ Repeat imaging is recommended in high-resource settings (e.g., USA^2^ or Germany^3^) and only conditionally recommended by the WHO on account of cost,^4^ which can limit access in low-resource settings.

Point-of-care ultrasound (POCUS) is a potentially portable, radiation-free bedside imaging modality that is emerging as integral part of TB diagnostic workup, particularly for HIV-associated extra-pulmonary TB (EPTB) in high-prevalence settings. Although data on the value of POCUS for TB treatment monitoring are scarce, some reports indicate that POCUS for PTB and EPTB can support TB treatment monitoring for detection of underlying problems like incorrect treatment, resistant TB, or non-TB diagnoses.^5–7^ For several EPTB manifestations, evidence remains anecdotal (e.g., thyroid TB^8^; pleural TB^9^). Considering lung ultrasound (LUS) for pulmonary TB (PTB), different sonographic features, such as small and large consolidations, B-lines, or effusions, may represent correlates of TB and are potential targets for sonographic monitoring^10–12^; however, available studies have not yet evaluated LUS patterns and their dynamics under ATT.^6,7,13^ Ultrasound appears conceivable as a treatment monitoring tool to detect issues with treatment adherence, drug resistance, or non-TB diagnoses, but the lack of data describing the evolution of ultrasound findings during ATT limits its implementation.

This study investigated pulmonary and extra-pulmonary ultrasound pathology in TB cases in India and Germany at baseline and following ATT initiation.

## METHODS

This was a prospective multi-centre diagnostic accuracy study at one Indian teaching hospital (Christian Medical College, Vellore) and three German university hospitals (Heidelberg, Frankfurt, Cologne). Detailed study procedures have been published along with ultrasound accuracy data, in the primary study publications.^11,12^ Consecutive adult inpatients and outpatients presumed to have TB with a positive four-symptom WHO TB screen were included. Patients were excluded if already on ATT >7 days.^14^ This explorative analysis included a consecutive subset of patients from the main cohort who were initiated on ATT and were willing to consent to at least one follow-up ultrasound exam.

### Procedures

Participants underwent a TB-focused baseline ultrasound examination before ATT assessing for pulmonary (B-lines, effusion, subpleural consolidations [SPCs] of <1 or ≥1 cm in 14 lung zones)^15^ and extra-pulmonary findings (pericardial, pleural, and abdominal effusions; abdominal and parasternal internal mammary region [IMN] lymphadenopathy and peripheral clinically presumed TB-lymphadenopathy; splenic or hepatic micro-abscesses; peritoneal or omental thickening). Follow-up ultrasound after ATT initiation was performed only on organs with baseline pathology, at the following time-points based on scheduled patient visits: T1 (1–2 months ± 14d), T2 (2–3 months ± 14d), T3 (3–5 months ± 14d), and T4 (6–8 months ± 14d). Organs without baseline pathology were not re-examined to minimise participant time burden. Participants were also asked about the evolution of their symptoms (cough, fever, weight loss, and night sweats) and the ATT regimen taken. The following ultrasound devices were used: Edge II (Fujifilm Sonosite, Japan) in Vellore and Heidelberg, Aplio 300 (Toshiba, Japan) and Acuson Juniper (Siemens, Germany) in Frankfurt, and HS40 (Samsung, South Korea) in Cologne. Ultrasound was performed and interpreted locally by trained non-radiologist clinicians specifically trained for this study (proficiency ensured by evaluating trial clips, details provided previously^11,12^); clips were recorded for all views and reviewed for this analysis (RW and SFW). Reviewers were blinded to clinical data but were aware of timing of ultrasound and previous pathology.

### Reference standard and case definitions

Confirmed TB was defined by an extended microbiological reference standard (eMRS) as any sputum, fluid, or tissue sample positive for *Mycobacterium tuberculosis complex* by culture or PCR. Unconfirmed TB was defined by a composite reference standard (CRS) as empirical ATT with documented clinical improvement.^11,12^

### Lung ultrasound

To grade the severity and extent of lung pathology over time, point values were attributed to findings taking into account their clinical relevance (e.g., large consolidations > small consolidations): SPC_≥1 cm_: 5 points; SPC_<1 cm_: 1 point; B-lines: 0.5 points; pleural fluid: 1 point. Values were added for all lung zones for an absolute (baseline score) and a normalised score (baseline score = 100%) to compare relative changes over time relative to baseline in percentages (details of score development in Supplementary Data). Also, an analysis of baseline scores for all presumed TB cases enrolled in the main accuracy study was performed stratified by smoking, post-TB history, HIV-status, and age, independent of TB status (see Supplementary Data).

### Extra-pulmonary ultrasound

For pleural effusion, presence or absence, laterality, and estimated volume were noted. Change was graded into worse (estimate >133% of baseline), similar (67%–133%), improved (<67% of baseline or change from bilateral to unilateral), or mixed. For enlarged lymph nodes, presence or absence and the size of the largest node were recorded. Follow-ups were graded based on the increase or decrease of maximum node sizes as worse (estimate >133% of baseline), similar (67%–133%), and improvement (<67% or change from bilateral to unilateral). For ascites, presence or absence and the operator-estimated volume (large, moderate, minimal) was recorded, as well as the number of quadrants involved and provided proportions per time-point. Presence or absence of peritoneal changes, the type of pathology (thickening, nodularity), location (parietal/omental), and the maximum thickness of pathology were recorded.

### Statistical analysis

Normalised LUS scores were visually depicted in spaghetti-plots and boxplots. The T test and χ² test were used to assess possible selection bias between participants under ATT who attended or did not attend follow-up visits. Differences in LUS scores between time-points were compared using a Wilcoxon signed rank test. Inter-observer agreement between initial and review ultrasound interpretation was assessed using Cohen’s kappa statistic for each lung zone and each finding (SPC_≥1 cm_, SPC_<1 cm_, B-lines, effusion) separately. Sample size calculation for the primary analysis does not apply to this secondary analysis. Missing data are indicated by denominators where applicable. Statistical analyses used R v.4.3.3 (packages: openxlsx, ggplot2, dplyr, gridExtra, irr, UpSet) and Stata (StataCorp. 2024. Stata Statistical Software: Release 17. College Station, TX: StataCorp LLC). RedCap (version 15.0.26^16^) was used for data collection.

### Ethical statement

This study was approved by the respective institutional review boards (Vellore: CMC IRB No: 14342; Heidelberg S-314/2021, Frankfurt 2021-466; Cologne 22-1009), registered in the German trial registry (DRKS00026636), and conducted according to Good Clinical Practice guidelines and the Helsinki Declaration. Written informed consent was obtained from all participants. This manuscript conforms to the STROBE reporting guidelines (Supplementary Data).^17^

## RESULTS

Between January 2022 and July 2023, 179 enrolled participants were positive as per eMRS or CRS and initiated ATT. Of these, 71 (40%) attended at least one follow-up visit. An overview of cohort characteristics is provided in Supplementary Data Table S2 along with a comparison between participants who did not attend follow-up visits, which showed no relevant differences between groups with the exception of the ultrasound findings pertaining to the *Focused assessment with sonography for HIV-associated tuberculosis* (FASH) ultrasound.

### Cohort description

**Figure 1.**
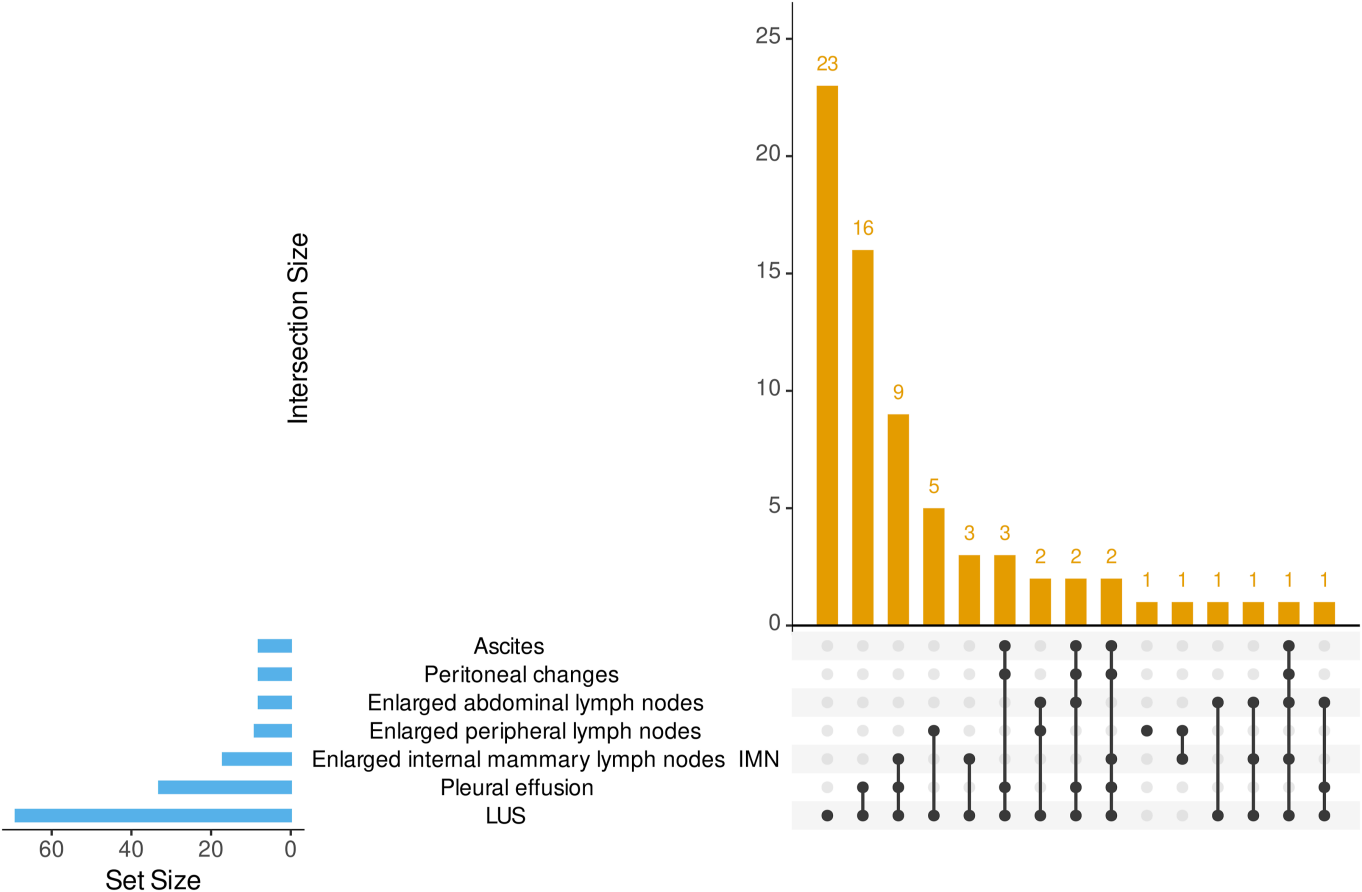
Upset plot for combinations of individual ultrasound findings. LUS = lung ultrasound scores positive; IMN = internal mammary lymph node.

Of 71 participants, 54 (76%) had microbiologically confirmed TB (eMRS), and 17 (24%) were diagnosed clinically (CRS). The most frequent ultrasound pathology was a positive LUS score (69 participants), followed by pleural effusions and enlarged internal mammary lymph nodes (IMNs) (33 and 17 participants, respectively). The reference standard distributions for individual ultrasound findings and their most frequent combinations are provided in Table 1 and Figure 1. The majority of participants (55/71, 77%) had drug-susceptible TB (DS-TB) treated with standard four-drug regimen; 8/71 (11%) had DS-TB treated with individual regimens (e.g., prolonged treatment phase); 5/71 (7%) had drug-resistant TB (DR-TB, n = 3 MDR, n = 2 isoniazid mono-resistance) and received appropriate regimens depending on drug susceptibility; 4/71 (6%) received individual regimens for unclear reasons. Self-reported symptoms showed improvement with ATT in 62/71 (87%), while 8/71 (11%) reported mixed changes of their symptoms (no improvement or mixed improvement and worsening), and 1/71 (1%) reported worsening of symptoms.

**Table 1.** Reference standards for all participants and individual ultrasound findings.

n (%)	All	LUS	Pleural effusion	Enlarged IMNs	Enlarged peripheral lymph nodes	Enlarged abdominal lymph nodes	Ascites	Peritoneal changes
CRS	17/71 (24)	16/69 (23)	12/33 (36)	6/17 (35)	3/9 (33)	2/8 (25)	3/8 (38)	3/8 (38)
eMRS	54/71 (76)	53/69 (77)	21/33 (64)	11/17 (65)	6/9 (67)	6/8 (75)	5/8 (63)	5/8 (63)
All	71	69	33	17	9	8	8	8

LUS = lung ultrasound score positive; IMN = internal mammary lymph node; CRS = composite reference standard; eMRS = extended microbiological reference standard.

### Lung ultrasound, n = 69

69 participants had at least one follow-up, 35 at least two, 17 at least three, and 5 had four follow-ups. Time-point results were available for n = 25 at T1, n = 49 at T2, n = 25 at T3, and n = 22 at T4. The most common finding before ATT was SPC_<1 cm_ (median 3 zones, interquartile range [IQR]: 2–5), followed by effusion (median 2 zones, IQR: 0–6), SPC_≥1 cm_ (median 2 zones, IQR: 0–3), and B-lines (median 1 zone, IQR: 0–2) (Table 2). The median absolute baseline LUS score was 15.5 (IQR: 7–27.5).

**Table 2.** Pulmonary ultrasound findings in TB cases under ATT, lung ultrasound scores.

		Pre-ATT	Post-ATT			
		Baseline, n = 69	T1 (1–2 months), n = 25	T2 (2–3 months), n = 49	T3 (3–5 months), n = 25	T4 (6–8 months), n = 22
Lung (n = 68)						
SPC_≥1 cm_	Median number of zones (IQR) with finding	2 (0; 3)	2 (0; 3)	1 (0; 2)	1 (0; 1)	0 (0; 1)
SPC_<1 cm_		3 (2; 5)	2 (1; 5)	3 (1; 6)	3 (1; 3)	2 (1; 5)
B-lines		1 (0; 2)	1 (0; 2)	0 (0; 2)	0 (0; 1)	0 (0; 0.75)
Effusion		2 (0; 6)	2 (0; 5)	1 (0; 4)	1 (0; 2)	1 (0; 2)
LUS score (5/1/0.5/1)	Median score (IQR)	15.5 (7; 27.5)	12 (3.5; 21.5)	11 (4.5; 23)	7 (3; 10)	7 (2.25; 10.38)
LUS score normalised (baseline = 1)	Median proportion of baseline score	NA	1 (0.66; 1.25)	0.86 (0.63; 1.02)	0.67 (0.37; 0.83)	0.47 (0.31; 0.67)
*P* value	Compared to baseline	NA	0.5841	0.0023	0.0008	0.0001

ATT = anti-TB treatment; SPC = subpleural consolidation; LUS = lung ultrasound; IQR = interquartile range.

### LUS score under ATT

**Figure 2.**
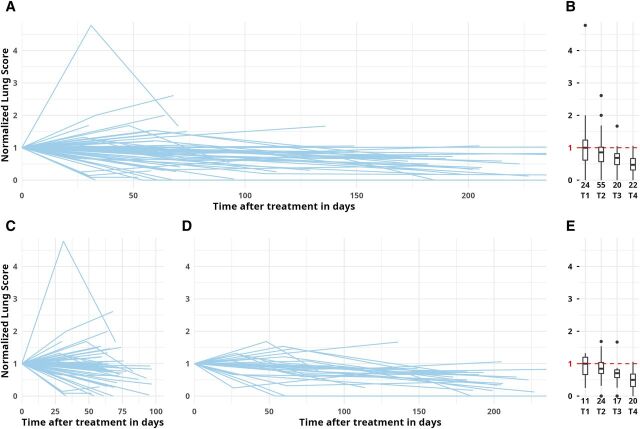
Normalised lung score under anti-TB treatment (ATT). **A, B:** Spaghetti and boxplots for all participants with any available time-points, n available datapoints. **C:** Spaghetti plot only for participants with at least T1 or T2 (early time-points). **D, E:** Spaghetti and boxplots only for participants with both early (T1 or T2) and late (T3 or T4) time-points, n available datapoints. LUS score: SPC_≥1 cm_: 5 points; SPC_<1 cm_: 1 point; B-lines: 0.5 points, effusion: 1 point, added sum of 14 lung zones; normalised for baseline value (=1.0). LUS = lung ultrasound; SPC = subpleural consolidation.

The median number of lung zones with findings decreased over time with SPC_<1 cm_ being the most common residual finding at T4 (median 2 zones). The plot of individual participant results showed a decline in normalised LUS score over time (Figure 2). At T1, a steady median score with a wide distribution (median 100%; IQR: 66–125) was observed. Two cases showed increases >200% of their baseline values (one uncomplicated clinical course, one case with increasing symptoms at follow-up). At the programmatically relevant time-point T2 (typically shift from intensive to continuous therapy) the LUS score was significantly lower that at baseline (86% compared to baseline, IQR: 63%–102%, *P* = 0.0023), and the decline steadily continued until T4 (median 47%, IQR: 31%–67%, *P* = 0.0001). Only one participant had a score of ≥100% of baseline at T4 (pre-XDR [extremely drug-resistant] TB with previous TB episodes). Including only participants with at least one early (T1/2) and one late (T3/4) time-point to reduce inclusion bias showed similar dynamics (Figure 2D and 2E).

LUS score trends of participants with self-reported improvement were lower at T1 than for those with mixed symptom evolution (median LUS score 98%, IQR: 48–118 vs. 184%, IQR: 145–270). This difference was not observed at T2, and few datapoints were available for T3/4 (Supplementary Data Figure S3A and S3B). LUS scores in participants on standard ATT versus non-standard ATT only were comparable at T1–3 but were lower at T4 in the standard therapy group (median LUS score 34%, IQR: 18–67 vs. 59%, IQR: 50–68; Supplementary Data Figure S3C and S3D). Inter-observer agreement between bedside ultrasound interpretation showed a kappa of 0.901 for SPC_<1 cm_, 0.914 for SPC_≥1 cm_, 0.805 for B-lines, and 0.876 for effusion.

### EPTB findings under ATT

The dynamics of EPTB ultrasound findings under ATT varied by organ system (Table 3; further details in Supplementary Data). Pleural effusions (n = 33) did not resolve quickly (10/10 cases had effusions at T1, Table 3), and many had residual effusions at T4 (8/12, 67%), but volume decreased continuously across time-points. For IMNs (n = 17), and peripheral and abdominal enlarged lymph nodes (n = 8 each), a decrease in size was seen in 86% of IMNs and 100% of peripheral and abdominal cases. Incomplete resolution (e.g., size above individual threshold) was observed in 57% of IMNs, 33% of peripheral and 0% of abdominal lymph nodes. Ascites (n = 8) showed rapid improvement with ATT and resolved in all cases observed at T4, whereas peritoneal changes (n = 8) also showed improvement with ATT, but changes persisted in 40% of observed cases at T4. Spleen lesions (n = 3) and pericardial effusions (n = 2) were not analysed due to small numbers.

**Table 3.** Extra-pulmonary TB (EPTB) ultrasound findings under ATT.

		Pre-ATT	Post-ATT			
		Baseline	T1 (1–2 months)	T2 (2–3 months)	T3 (3–5 months)	T4 (6–8 months)
Pleural effusion (n = 33)						
Present?	n, %	33/33 (100)	10/10 (100)	19/24 (79)	3/7 (43)	8/12 (67)
Volume estimate	Median (IQR) % of baseline	NA	101 (63; 159) (n = 10)	36.5 (11; 69) (n = 24)	25 (0; 84) (n = 5)	29.5 (7; 68) (n = 9)
Improvement (I), similar (S), worse (W), mixed (M) compared to baseline	n, %	NA	I 3 (30), S 3 (30), W 3 (30), M 1 (10) (n = 10)	I 20 (83), S 2 (8), W 2 (8) (n = 24)	I 4 (80), S 1 (20) (n = 5)	I 8 (89), S 1 (11) (n = 9)
Internal mammary lymph nodes ≥ 0.5 cm (n = 17)						
Present?	n, %	17/17 (100)	5/7 (71)	6/9 (67)	2/7 (29)	4/7 (57)
Improvement (I), similar (S), worse (W), mixed (M) compared to baseline	n, %	NA	I 4 (57), S 3 (43) (n = 7)	I 4 (44), S 5 (56) (n = 9)	I 5 (71), S 2 (29) (n = 7)	I 6 (86), S 1 (14) (n = 7)
Peripheral lymph nodes (n = 9)^A^						
>1.5 cm present?	n, %	9/9 (100)	3/4 (75)	5/5 (100)	5/7 (71)	2/6 (33)
Decline in max. size compared to baseline?	n, %	NA	2/4 (50)	5/5 (100)	7/7 (100)	6/6 (100)
Max. size compared to baseline	Median (IQR) %	NA	87.5 (69;105) (n = 4)	76 (62;88.5) (n = 5)	57.5 (49;62) (n = 7)	60.5 (52;65) (n = 6)
Abdominal lymph nodes ≥ 1.5 cm (n = 8)						
Present?	n, %	8/8 (100)	2/3 (67)	0/5 (0)	1/4 (25)	0/3 (0)
Ascites (n = 8)						
Present?	n, %	8/8 (100)	3/4 (75)	3/5 (60)	1/3 (33)	0/5 (0)
Peritoneal changes (n = 8)^B^						
Present?	n, %	8/8 (100)	4/4 (100)	3/5 (60)	2/3 (67)	2/5 (40)

ATT = anti-TB treatment; IQR = interquartile range.

A For median calculation, measurements <1.5 cm counted as % just below 1.5 cm with baseline size as reference.

B Nodular or laminar peritoneal thickening observed in 3/8 (38%) each; omental thickening: of seven cases with omental thickening, all showed improvement over time: 4/7 (57%) showed complete resolution and 3/7 (43%) showed decrease in max. thickness over time. Nodules: three cases with nodular thickening showed diverse phenomena: one case with nodules (max. 4 mm) showed improvement at T3 (resolution of nodules, did not attend T1/2). Another showed improvement (>5 nodules, max. 3 mm) with a reduced number at T1 (2–5 nodules, max. 2 mm; no further follow-ups). The third had multiple nodules at t0 (>5, max. 3 mm), that mostly resolved at T2, but a single nodule persisted and increased in size (T2 2.5 cm, T3 3.6 cm, T4 3 cm). Peritoneal laminar thickening: all three cases of laminar peritoneal thickening showed improvement over time with either decrease in max. thickening or complete resolution.

## DISCUSSION

This prospective Indian and German study is one of the largest to investigate changes in pathology under ATT using ultrasound and the first to introduce a systematic LUS score to quantify TB pathology over time. LUS scores showed increases and decreases early after therapy initiation, followed by a steady decline, reaching a median of 47% of baseline score after 6–8 months of ATT. Initial increases in LUS scores were seen in participants with clinical improvement and therefore may represent a paradoxical reaction rather than indicate therapy failure. Persisting lesions despite clinical symptom resolution may represent TB sequelae. Stratification of LUS score dynamics by symptom evolution showed a correlation mostly at T1, suggesting that LUS may detect early deterioration, for example, during a paradoxical reaction. These trends should be assessed in future studies for their utility in informing treatment decisions. Regarding other potential tools to quantify lung pathology with ultrasound, one opinion paper suggested a comparable lung score comprising B-lines and consolidations for lung aeration in pneumonia in the ICU.^18^ A TB-specific LUS score has not been proposed yet, but this study expands on the aforementioned score by incorporating TB features like pleural effusion and apical consolidations.

Comparable longitudinal data in adults with TB are sparse. One study followed up patients with pneumonia, but only a subset had TB and follow-up was one only week.^13^ Persistence of LUS pathology at 6 months was also reported by paediatric cohorts, where persistent TB consolidations did not correspond with treatment failure.^6,7^ In post-COVID lung disease, LUS has also demonstrated persisting signs like B-lines despite clinical recovery.^19^ It remains unclear if persistent findings on LUS may be due to incomplete healing processes, permanent scarring (TB sequelae), or background pathology in the population unrelated to the TB episode. Chest X-ray (CXR) response criteria have been proposed,^20^ and, notably, persistent CXR pathology during ATT and even beyond cure is frequently seen in TB survivors, suggesting post-TB sequelae.^21^ The prevalence of radiographic abnormalities after ATT completion was 46% in one meta-analysis, in line with our ultrasound data.^22^ PET-CT imaging has also revealed persistent metabolic activity in affected tissues even after microbiological cure, suggesting ongoing inflammation at the end of treatment.^23^

Similar to LUS, EPTB ultrasound showed trends of improvement under ATT, as well as persistence of some residual findings. While our case numbers were low, the overall dynamics of EPTB ultrasound pathology in this cohort were comparable with other cohorts showing improvement under ATT.^5–7^ The difference in FASH-positive cases between participants with and without follow-up likely reflects the higher proportion of EPTB in the German cohort^11,12^ and better follow-up adherence in that setting. Data on EPTB sequelae are scarce, but a review suggests residual organ dysfunctions to be a common feature for abdominal, urogenital, spinal, and cutaneous TB.^24^

In pleural effusions, presence or absence alone may underestimate relevant changes, but volumetric measurements, which showed gradual improvement over time in most cases, may be a more reliable indicator of response/failure. Persistent effusions seen in our study were in line with previous data (15%–41% after 6 months of ATT).^25^ Initial volume increases have been described in a significant proportion of TB pleural effusion and represent a paradoxical reaction.^26^

Dynamics in abdominal TB varied. While ascites resolved rapidly in most cases, omental changes like thickening improved more gradually, which is also described in case reports or case series.^27,28^ For participants with enlarged abdominal, peripheral, and thoracic (IMNs) lymph nodes, most showed gradual improvement (few cases had an initial increase in size), but occasionally a size under the cut-off was not achieved at the end of ATT (e.g., peripheral lymphadenopathy in 33% at T4). Persistence of enlarged lymph nodes after ATT has been observed in approximately 20% of patients with peripheral lymph node TB but was not associated with treatment failure.^29^ The application of a combined LUS and EPTB scoring system may be considered in future research. However, the small number of EPTB findings in our cohort limited this approach.

Limitations include the fact that although baseline characteristics between the follow-up cohort and those not followed up were comparable, only 40% of eligible participants attended follow-up visits, which limits generalisability due to possible selection bias. Some cases without documented improvement in early visits were not available for further imaging so later improvement may have been missed. Insufficient data on failed treatment courses limit conclusions for ultrasound in treatment failure. For LUS, no validated metric exists for quantifying and monitoring disease severity in TB and inclusion of a healthy control group would have helped to define background pathology relative to the general population. However, the LUS score enabled a standardised measure integrating commonly assessed LUS findings and may thus be replicated or adapted to future POCUS research. For rarer EPTB findings, which we assessed using exploratory metrics and thresholds, data are limited to case series. Retrospective approaches may enable more adequate sample sizes and the exploration of robust metrics. Nevertheless, inclusion of detailed EPTB assessments provides insight into dynamics that would otherwise be limited to case reports. A strength of this study was the excellent inter-observer agreement between bedside operators and secondary reviewers, suggesting the feasibility of ultrasound training to non-radiologist clinicians.

## CONCLUSION

Our exploratory study provides evidence for the already prevalent practice of ultrasound in TB treatment monitoring, and the LUS score may serve as a marker of disease severity. While sonographic pathology showed variable early dynamics, most participants showed gradual improvement with ATT. The significance of incomplete resolution after treatment remains uncertain, and there is a need for further research on TB sequelae. Ultrasound is a non-invasive, radiation-free technology with the potential as an accessible tool for TB treatment monitoring, particularly in settings with limited access to other monitoring tools.

## Supplementary Material




