# Supplementary material for: Ultrasound dynamics during treatment of pulmonary and extra-pulmonary TB
Source: IJTLD Open. 2026 Mar 13;3(3):169–76. doi: 10.5588/ijtldopen.25.0608 (PMC12991774; doi:10.5588/ijtldopen.25.0608)
Supplement: Supplementary file 1 [file ijtldopen25-0608_supplementarydata1.pdf]

SUPPLEMENT for

Ultrasound dynamics during treatment of pulmonary and extrapulmonary tuberculosis in India and Germany

Contents

1. Exploratory development and choice of LUS score with Supplement Figure 1
2. Supplementary results and discussion: baseline data in presumed TB
- Supplement Methods
- Supplement Results
- Supplement Discussion
- Supplement Table 2: Baseline LUS Score in presumed TB cases with subgroup analyses
- Supplement Figure 2: Baseline lung ultrasound score in presumed TB cases with subgroup analyses
3. Supplement Table 1: Baseline and follow-up population
4. Supplement Figure 3 – LUS scores stratified by symptom evolution and ATT regimen
5. Supplementary Results: further details of EPTB findings under ATT
6. STROBE Statement

1. Exploratory development and choice of LUS score

See Supplement Figure 1.

- Consolidations > and < 1cm should be included, larger=more relevant, but how much?)
    - o if „C“onsolidation (>=1cm) = „S“mall consolidation (<1cm) = 1 (C1/S1/B0/I0/E0): smaller difference in reduction
    - o if C=20, then even 14 zones with S would be irrelevant, also extreme outliers (C20/S1/B0/I0/E0 and C10/S1)
    - o C5/S1 and C3/S1: fewer outliers
- Adding „B“-lines (less relevant than suns or same relevance?)
    - o C5/S1/B0.5 less outliers at t4 evenly divided than C5/S1/B1
    - o C5/S1/B0.5 cleaner temporal trend than C3/S1/B0.5
- Taking „E“ffusion into account
    - o C5/S1/B0.5/E0 vs C5/S1/B0.5/E1: E1 fewer outliers, no relevant change for t4, but smaller score distribution → may cover early changes better
    - o E1 vs E2, no relevant differences, but slightly smaller score distribution → favor E1
- Final score: **C5/S1/B0.5/E1**

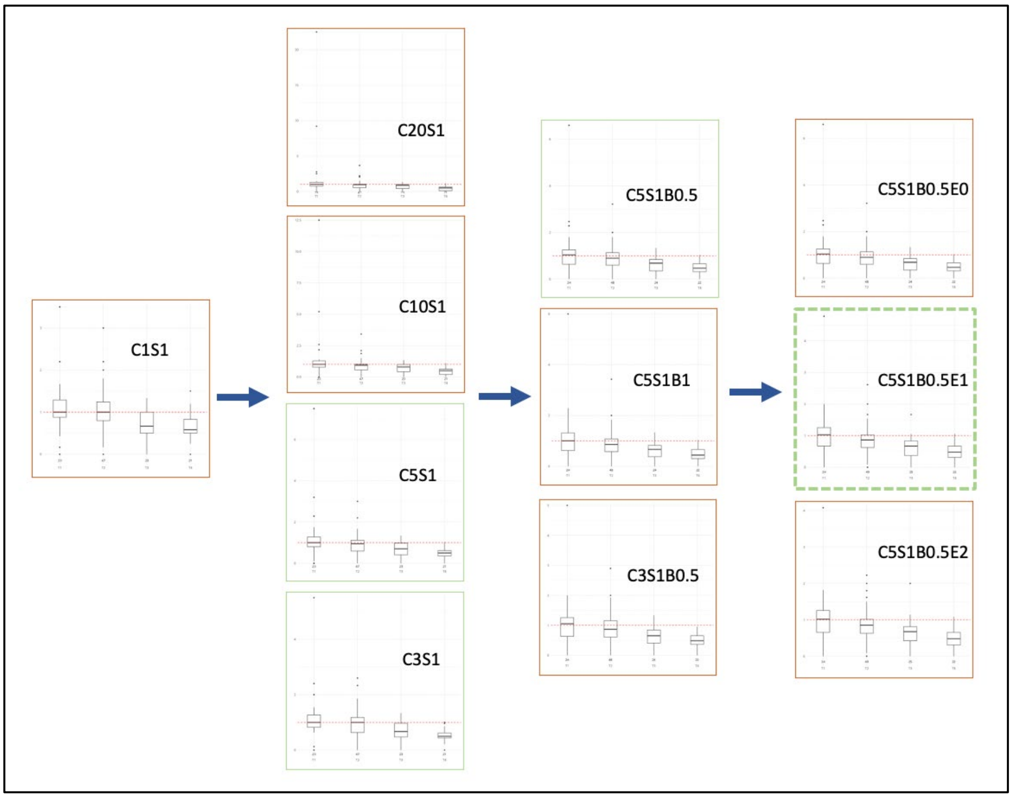

Supplement Figure 1, explorative score development
Comparing different weights for „C“onsolidation (>=1cm) = „S“mall consolidation (<1cm), “B”-lines and “E”ffusion, each counted individually for each of the 14 lung zones.

Red frame: discarded, green frame: further explored, green dashed frame: final score used

2. Supplementary results: baseline data in presumed TB

Supplement Methods

Data analysis was conducted using Stata (StataCorp. 2024. Stata Statistical Software: Release 17. College Station, TX: StataCorp LLC.).

Supplement Table 1: comparison of TB cases that attended follow-up visits, differences were evaluated with t-test for continuous variables and chi-square test for categorical variables.

Supplemental Table 2: Linear regression was used to test if participant characteristics significantly predicted baseline ultrasound score. Characteristics with a p-value <0.1 in the univariate analysis were included in the multivariable model, and retained in the model if the p value was <0.05.

Figure S2. Test difference in medians with ranksum

Supplement Results

For the baseline cohort (n = 647 participants with presumed TB, [Weber et al. OFID 2024, Weber et al. PLOS One 2025]), the median number of lung zones with $SPC_{>=1cm}$, $SPC_{<1cm}$, B-lines and effusion and the LUS-score per participant is provided in Supplement Table 1. The most common finding was $SPC_{<1cm}$ (median 3 zones affected). The median calculated LUS score was 10 (IQR 3;22.5).

LUS scores were compared across the variables TB status, HIV status, sex, age, post-TB status, smoking status, diabetes status and country of recruitment (Supplement Table 2 and Figure 2a). The variable post-TB status had the strongest effect with higher LUS scores in participants who had previous TB episodes. Overall, TB cases had higher scores than non-TB cases (14.5 vs 8.5) and within TB cases, those with a clinical diagnosis had higher scores than confirmed cases (24.5 vs 12).

Data stratified by TB status is provided in Figure 2b and 2c. Within TB cases, LUS scores were higher for participants with clinical TB diagnosis, post-TB status, smoking and those who were recruited in India. In non-TB cases, higher LUS scores were seen for participants with higher age and post-TB status.

TB disease, male gender, age older than 46 years, history of previous TB disease, and participants at the India site were all significantly associated with higher baseline ultrasound scores (Supplement Table 2).

Supplement Discussion

Despite higher median LUS scores among TB cases compared to non-TB cases, there was substantial overlap, limiting the score's potential diagnostic utility due to low specificity. Interestingly, participants with a clinical TB diagnosis had higher LUS scores than those with confirmed TB. Possibly this reflects a greater degree of radiologically visible lung pathology resulting in empirical treatment.

Post-TB status was consistently associated with higher LUS scores, including in participants without current TB, indicating that LUS abnormalities may persist after successful TB treatment and likely represent structural lung sequelae rather than active infection. This may be an explanation for persisting LUS scores after complete ATT (cf. main manuscript).

Country-level comparison showed higher LUS scores among Indian TB cases, while scores in non-TB cases were comparable to those from Germany. This may reflect more advanced disease at presentation in India, possibly due to delayed diagnosis (in Germany participants were frequently diagnosed through active migrant screening) or selection bias through recruitment in tertiary care center.

In summary, the LUS score may be a tool to assess disease severity, but appears of limited value in diagnosis, with post-TB status being the most relevant factor to increase the score.

Supplement Table 2, Baseline LUS Score in presumed TB cases with subgroup analyses

	n	Median zones with finding (IQR)				Median LUS score (IQR)	p-value for median [#]	Univariate [§]		Multivariable regression [&]	
		SPC <1cm	SPC >=1cm	B-lines	Effusion			Effect size (beta), 95% CI	p-value	Effect size (beta), 95% CI	p-value
All	647	3 [1;6]	1 [0;2]	1 [0;2]	1 [0;4]	10 [3;22.5]			-		-
Unlikely TB	468	3 [1;6]	0 [0;2]	0 [0;2]	0 [0;3.25]	8.5 [2;20]	0.002	2.34 (0.33, 4.36)	0.023	5.37 (3.25, 7.49)	<0.001
TB (confirmed and clinical)	179	3 [2;5]	1 [0;3]	1 [0;3]	1 [0;4]	14.5 [5.75;25]					
Clinical TB	32	2 [1;4]	3 [0;4]	0.5 [0;2]	6 [1;7]	24.5 [6.75;30]	0.107	-4.09 (-8.28, 0.09)	0.055	-	-
Confirmed TB	147	4 [2;5]	1 [0;3]	1 [0;3]	1 [0;4]	12 [6.25;24]					
HIV-negative	613	3 [1;6]	1 [0;2]	1 [0;2]	1 [0;4]	10 [3;22.5]	0.569	0.58 (-5.88, 7.05)	0.860	-	-
HIV-positive	13	4 [3;7]	1 [0;2]	2 [1;5]	0 [0;2]	15 [4.5;22.5]					
Female	210	3 [1;5]	0 [0;2]	0 [0;2]	0 [0;2.75]	8.25 [2;18.38]	0.002	2.68 (0.76, 4.61)	0.006	2.31 (0.48, 4.15)	0.014
Male	437	3 [1;6]	1 [0;2]	1 [0;3]	1 [0;4]	10.5 [3;18.25]					
*Age <=46 years**	334	3 [1;5]	0 [0;2]	0.5 [0;2]	0 [0;3]	8 [2;19.38]	<0.001	4.11 (2.33, 5.89)	<0.001	3.96 (2.21, 5.70)	<0.001
Age >46 years	313	4 [2;6]	1 [0;3]	1 [0;3]	1 [0;5]	12 [4.5;24.5]					
No post-TB	536	3 [1;5]	0 [0;2]	0 [0;2]	0 [0;3]	8.5 [2;18.62]	<0.001	7.52 (5.16, 9.88)	<0.001	7.68 (5.38, 9.98)	<0.001
Post-TB	108	5 [3;8]	2 [1;3.25]	1 [0;3.25]	2 [0;6]	21 [10;30.12]					
Non-smoker	484	3 [1;5.25]	0 [0;2]	0 [0;2]	0 [0;4]	9 [2;22]	0.058	1.00 (-1.07, 3.07)	0.342	-	-
Smoker	163	4 [2;6]	1 [0;2]	1 [0;3]	1 [0;4]	11.5 [4.5;23]					
<5 packyears	44	3 [1.75;6]	0 [0;2]	1 [0;2]	0.5 [0;3.25]	8.5 [3.75;19.12]	(ref)	(reference)	-	NA	NA
5-10 packyears	32	4.5 [2;6]	1 [0;3.25]	2.5 [0.75;4]	1.5 [0;4]	13.25 [5.25;25.88]	0.245	1.33 (-3.48, 6.14)	0.585		
10-15 packyears	24	4 [2.75;6]	1 [0;2.25]	1 [0;4]	1.5 [0;4]	14.75 [8.88;22.75]	0.147	2.22 (-3.03, 7.47)	0.406		
>15 packyears	63	3 [1;6]	1 [0;2]	1 [0;3]	1 [0;2.5]	11.5 [4.75;21.5]	0.768	-0.41 (-4.48, 3.65)	0.842		
Non-diabetic	449	3 [1;6]	1 [0;2]	1 [0;2]	0 [0;4]	10 [2;23]	0.330	0.70 (-1.59, 2.99)	0.547	-	-
Diabetic	136	3 [1;5]	1 [0;2.25]	0 [0;2]	2 [0;4]	10.75 [4;23.75]					
Germany	103	4 [1;5]	1 [0;2]	2 [1;4]	0 [0;2]	8.5 [3.5;16.5]	0.076	3.05 (0.58, 5.51)	0.015	4.43 (1.79, 7.07)	0.001
India	544	3 [1;6]	1 [0;2]	0 [0;2]	1 [0;4]	10.5 [3;23.5]					

TB tuberculosis; IQR interquartile range; LUS lung ultrasound; SPC subpleural consolidation; HIV human immunodeficiency virus

* 46 years = median age
[#] Wilcoxon rank-sum test
[§] linear regression
[&] only significant variables from univariate analysis included in model

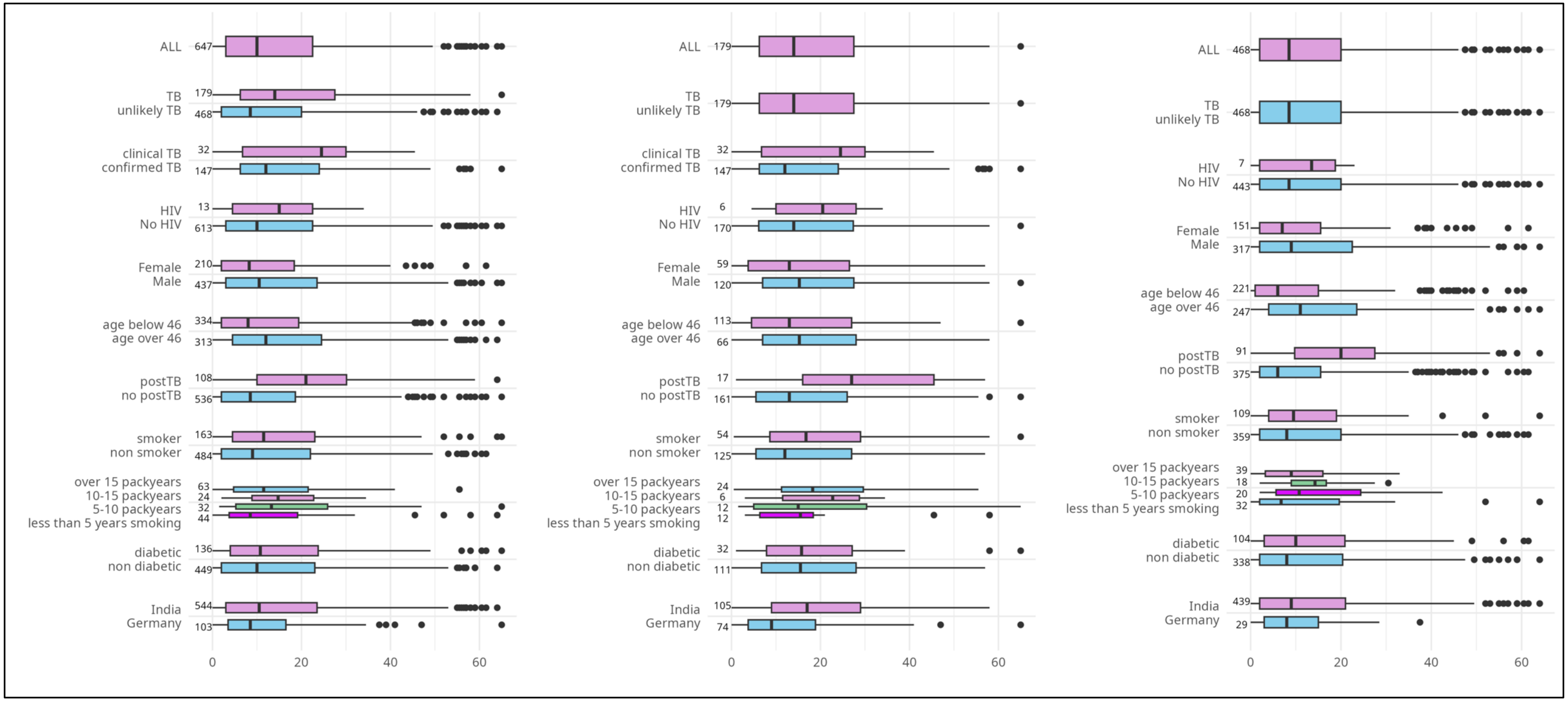

Supplement Figure 2

Baseline (absolute) lung ultrasound score for all participants and subgroups

left: All participants
center: Only TB-cases
right: Only unlikely TB cases

3. Supplement Table 1 Baseline and follow-up population

	TB cases not followed up	Follow-up cohort	p-value
n=177	106	71	
Age in years	43 [29;58] (N=106)	40 [31;51] (N=71)	0.438
Gender male	70/106 (66%)	48/71 (68%)	0.829
Body mass index (kg/m^2)	20.4 [17.9;24.2] (N=105)	20.9 [19;23.8] (N=71)	0.408
History of tobacco use *	35/106 (33%)	20/71 (28%)	0.496
Final diabetes status	18/84 (21%)	14/58 (24%)	0.640
Final HIV status	2/105 (2%)	4/69 (6%)	0.170
Cough	85/106 (80%)	58/71 (82%)	0.804
Night sweats	30/106 (28%)	20/71 (28%)	0.985
Fever	56/104 (54%)	35/70 (50%)	0.859
Weight loss	72/104 (69%)	48/70 (69%)	0.967
C-reactive protein (mg/l)	19 [4;38] (N=98)	20 [5;50] (N=68)	0.683
Hemoglobin (g/dl)	12 [10;14] (N=103)	12 [11;14] (N=70)	0.455
CXR suggestive of active TB	26/83 (31%)	19/56 (34%)	0.823
FASH (original) positive	43/106 (41%)	39/69 (57%)	0.031
Subpleural consolidations (SPC) <1cm present	94/106 (89%)	62/69 (90%)	0.991
Subpleural consolidations >=1cm present	65/106 (61%)	49/69 (71%)	0.296

Interpretation: There were no significant differences between participants who returned for follow-up visits and those who did not, except for FASH results. Those who were FASH-positive were significantly more likely to return for follow-up than those who were FASH-negative (p=0.03)

4. Supplement Figure 3 – LUS scores stratified by symptom evolution and ATT regimen

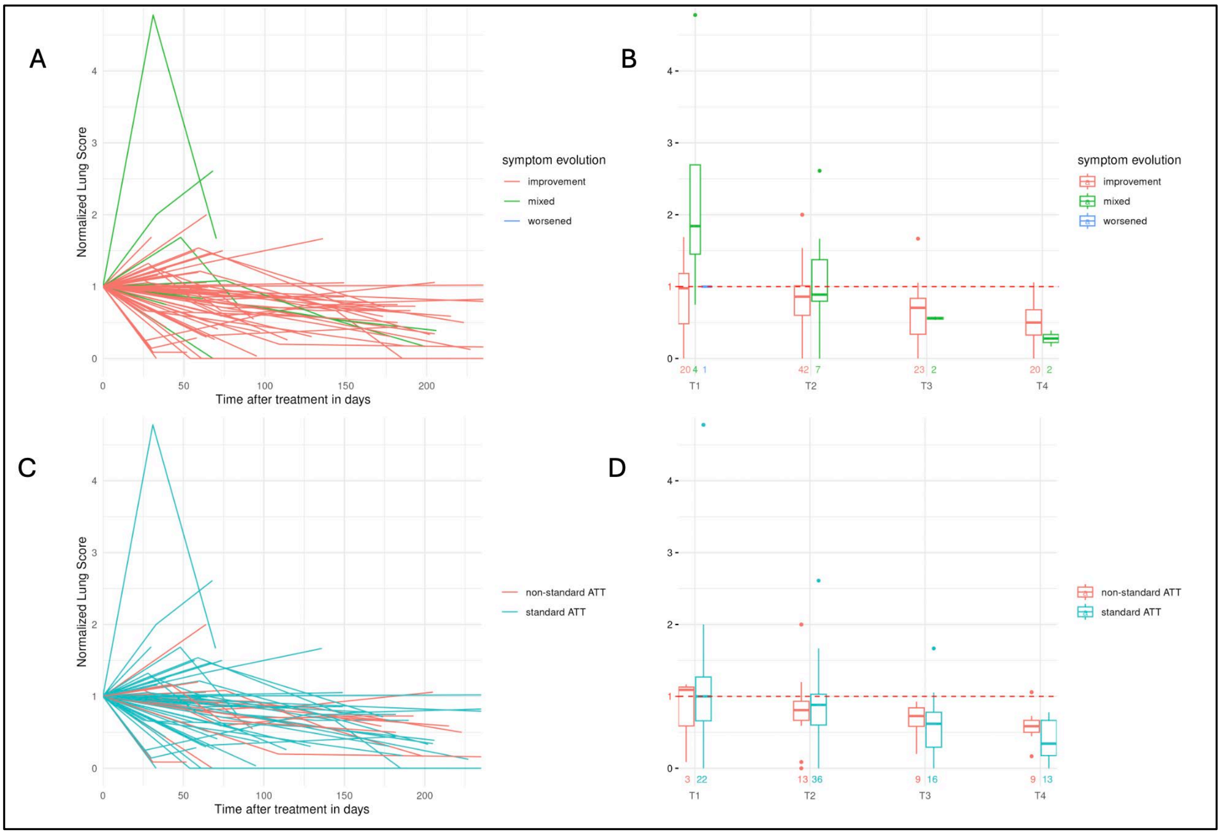

Supplement Figure 3
LUS scores stratified by symptom evolution and ATT regimen

A, B spaghetti plot and boxplot of normalized LUS score stratified by improved, mixed or worsening symptom evolution
C, D spaghetti plot and boxplot of normalized LUS score stratified by standard ATT and deviation from standard ATT

LUS lung ultrasound, ATT anti-TB treatment

5. Supplement Results: further details of EPTB findings under ATT

- *Pleural effusion, n=33*

Pleural effusions did not resolve quickly (10/10 cases had effusions at T1, Table 1b) and the majority had small residual effusions at T4 (8/12, 67%). However, a steady decline in volume across time-points was observed although small numbers were available for T3/T4. Integrating volume and laterality, the initial observations showed a worsening of mixed dynamic in 4/10 (40%) of cases, but after T2, most cases showed improvement (e.g., 8/9, 89% at T4).

- *Enlarged internal mammary lymph nodes (IMN), n=17*

IMNs >5mm persisted in 4/7 (57%) at T4, but taking into account decrease in size and laterality, improvement was seen in 86% at T4.

- *Enlarged peripheral lymph nodes, n=9*

Enlarged peripheral lymph nodes were present in the cervical (n=7), axillary region (n=1) or disseminated (n=1). At T1 2/4 (50%) showed a similar or slightly larger size than baseline, but a steady decrease in median size was subsequently seen across all cases. Complete resolution (<1.5cm cut-off) was documented only in 6/9 (67%) cases - 1/6 (17%) at T1; 2/6 (33%) at T3; and 3/6 (50%) at T4.

- *Enlarged abdominal lymph nodes, n=8*

In 6/8 (75%) only one region was affected, in 2/8 (25%) more than one region (5/8, 63% portal; 4/8, 50% para-aortic, 2/8, 25% in the right lower quadrant). Improvement in size or resolution were seen in all cases (earliest time-point: T1 in 2/8 (25%), T2 in 3/8 (38%) and T3 in 3/8 (38%) cases).

- *Ascites, n=8*

Ascites was present in eight participants in a median of 3.5 (IQR 3;4) abdominal quadrants and all cases were associated with peritoneal changes and in 2/8 (25%) with abdominal lymphadenopathy). Improvement (complete resolution or fewer quadrants) was rapid and documented for all participants. The earliest improvement occurred at T1 (n=4, 50%), T2 (n=2, 25%) and T3 (n=2, 25%).

- *Peritoneal changes, n=8*

Omental thickening was the most common sonographic finding for peritoneal TB (7/8, 88%). Initially, all cases had concurrent ascites and 2/8 (25%) had concurrent abdominal lymphadenopathy. The overall presence of at least one peritoneal finding decreased over time, but some changes persisted.

6. STROBE Statement—checklist of items that should be included in reports of observational studies

	Item No	Recommendation	Page No
**Title and abstract**	1	(*a*) Indicate the study's design with a commonly used term in the title or the abstract	2 (abstract)
		(*b*) Provide in the abstract an informative and balanced summary of what was done and what was found	2
**Introduction**			
Background/rationale	2	Explain the scientific background and rationale for the investigation being reported	3
Objectives	3	State specific objectives, including any prespecified hypotheses	3
**Methods**			
Study design	4	Present key elements of study design early in the paper	4
Setting	5	Describe the setting, locations, and relevant dates, including periods of recruitment, exposure, follow-up, and data collection	4
Participants	6	(*a*) *Cohort study*—Give the eligibility criteria, and the sources and methods of selection of participants. Describe methods of follow-up<br>*Case-control study*—Give the eligibility criteria, and the sources and methods of case ascertainment and control selection. Give the rationale for the choice of cases and controls<br>*Cross-sectional study*—Give the eligibility criteria, and the sources and methods of selection of participants	4
		(*b*) *Cohort study*—For matched studies, give matching criteria and number of exposed and unexposed<br>*Case-control study*—For matched studies, give matching criteria and the number of controls per case	NA
Variables	7	Clearly define all outcomes, exposures, predictors, potential confounders, and effect modifiers. Give diagnostic criteria, if applicable	5
Data sources/ measurement	8*	For each variable of interest, give sources of data and details of methods of assessment (measurement). Describe comparability of assessment methods if there is more than one group	5-6
Bias	9	Describe any efforts to address potential sources of bias	6
Study size	10	Explain how the study size was arrived at	6
Quantitative variables	11	Explain how quantitative variables were handled in the analyses. If applicable, describe which groupings were chosen and why	6
Statistical methods	12	(*a*) Describe all statistical methods, including those used to control for confounding	6
		(*b*) Describe any methods used to examine subgroups and interactions	Supplement
		(*c*) Explain how missing data were addressed	6
		(*d*) *Cohort study*—If applicable, explain how loss to follow-up was addressed	4

		Case-control study—If applicable, explain how matching of cases and controls was addressed<br>*Cross-sectional study*—If applicable, describe analytical methods taking account of sampling strategy	
		(*e*) Describe any sensitivity analyses	NA
**Results**			
Participants	13*	(a) Report numbers of individuals at each stage of study—eg numbers potentially eligible, examined for eligibility, confirmed eligible, included in the study, completing follow-up, and analysed	7 and original study
		(b) Give reasons for non-participation at each stage	Original studies
		(c) Consider use of a flow diagram	Original studies
Descriptive data	14*	(a) Give characteristics of study participants (eg demographic, clinical, social) and information on exposures and potential confounders	Table 1, Supplement Table 1
		(b) Indicate number of participants with missing data for each variable of interest	Denominators
		(c) *Cohort study*—Summarise follow-up time (eg, average and total amount)	Figure 1
Outcome data	15*	*Cohort study*—Report numbers of outcome events or summary measures over time	Table 2, Figure 1
		Case-control study—Report numbers in each exposure category, or summary measures of exposure	NA
		Cross-sectional study—Report numbers of outcome events or summary measures	NA
Main results	16	(*a*) Give unadjusted estimates and, if applicable, confounder-adjusted estimates and their precision (eg, 95% confidence interval). Make clear which confounders were adjusted for and why they were included	Table 2, Figure 1
		(*b*) Report category boundaries when continuous variables were categorized	NA
		(*c*) If relevant, consider translating estimates of relative risk into absolute risk for a meaningful time period	NA
Other analyses	17	Report other analyses done—eg analyses of subgroups and interactions, and sensitivity analyses	NA
**Discussion**			
Key results	18	Summarise key results with reference to study objectives	9-10
Limitations	19	Discuss limitations of the study, taking into account sources of potential bias or imprecision. Discuss both direction and magnitude of any potential bias	10-11
Interpretation	20	Give a cautious overall interpretation of results considering objectives, limitations, multiplicity of analyses, results from similar studies, and other relevant evidence	11
Generalisability	21	Discuss the generalisability (external validity) of the study results	10-11
**Other information**			
Funding	22	Give the source of funding and the role of the funders for the present study and, if applicable, for the original study on which the present article is based	12
